# Strain-Dependent Morphology of Reactive Astrocytes in Human- and Animal-Vole-Adapted Prions

**DOI:** 10.3390/biom13050757

**Published:** 2023-04-27

**Authors:** Rosalia Bruno, Geraldina Riccardi, Floriana Iacobone, Flavia Chiarotti, Laura Pirisinu, Ilaria Vanni, Stefano Marcon, Claudia D’Agostino, Matteo Giovannelli, Piero Parchi, Umberto Agrimi, Romolo Nonno, Michele Angelo Di Bari

**Affiliations:** 1 Department of Food Safety, Nutrition and Veterinary Public Health, Istituto Superiore di Sanità, 00161 Rome, Italy; 2 Reference Center for the Behavioral Sciences and Mental Health, Italian National Institute of Health, 00161 Rome, Italy; 3 IRCCS Istituto delle Scienze Neurologiche di Bologna, 40139 Bologna, Italy; 4 Department of Biomedical and Neuromotor Sciences (DIBINEM), University of Bologna, 40138 Bologna, Italy

**Keywords:** prion diseases, prion strains, bank vole, reactive astrocytes, morphology of reactive astrocytes, prion-like diseases, neurodegenerative diseases

## Abstract

Reactive astrogliosis is one of the pathological hallmarks of prion diseases. Recent studies highlighted the influence of several factors on the astrocyte phenotype in prion diseases, including the brain region involved, the genotype backgrounds of the host, and the prion strain. Elucidating the influence of prion strains on the astrocyte phenotype may provide crucial insights for developing therapeutic strategies. Here, we investigated the relationship between prion strains and astrocyte phenotype in six human- and animal-vole-adapted strains characterized by distinctive neuropathological features. In particular, we compared astrocyte morphology and astrocyte-associated PrP^Sc^ deposition among strains in the same brain region, the mediodorsal thalamic nucleus (MDTN). Astrogliosis was detected to some extent in the MDTN of all analyzed voles. However, we observed variability in the morphological appearance of astrocytes depending on the strain. Astrocytes displayed variability in thickness and length of cellular processes and cellular body size, suggesting strain-specific phenotypes of reactive astrocytes. Remarkably, four out of six strains displayed astrocyte-associated PrP^Sc^ deposition, which correlated with the size of astrocytes. Overall, these data show that the heterogeneous reactivity of astrocytes in prion diseases depends at least in part on the infecting prion strains and their specific interaction with astrocytes.

## 1. Introduction

Prion diseases or transmissible spongiform encephalopathies (TSEs) are fatal neurodegenerative disorders affecting humans and animals. Human prion diseases comprise several major phenotypes, namely Creutzfeldt–Jakob disease (CJD), Gerstmann–Sträussler–Scheinker disease (GSS), fatal familial insomnia (FFI), kuru, and variably protease-sensitive prionopathy (VPSPr) [1]. In animals, TSEs include classical and atypical scrapie in sheep and goats, classical and atypical bovine spongiform encephalopathy (BSE) forms in cattle, chronic wasting disease (CWD) in cervids, and camel prion disease (CPrD) in dromedary camels, as recently reported [2].

TSEs are characterized by the accumulation of PrP^Sc^, the pathological form of host-encoded prion protein (PrP^C^), considered to be the main or sole component of the infectious agent. According to the “protein-only” model, PrP^Sc^ self-propagates by an autocatalytic mechanism involving binding to PrP^C^ and templating the conversion of the latter protein to the PrP^Sc^ state [3]. This pathological isoform acquires high aggregation propensity leading to PrP^Sc^ deposition in the brain tissue, accompanied by spongiform change, astrogliosis, and neuronal loss.

Prion diseases may be caused by prion exposure (acquired forms), mutations in the *PRNP* gene (genetic or hereditary forms), and sporadic events in which the source of infection has not yet been demonstrated (idiopathic or sporadic forms). For instance, CJD, the most common human prion disease, occurs as sporadic (sCJD), genetic (gCJD), iatrogenic (iCJD), and variant CJD (vCJD) forms [1].

Although devoid of nucleic acids, prions occur as different strains. Strains are currently explained as different self-propagating conformational variants of PrP^Sc^, which impart their conformations to the host PrP^C^. The experimental infection of animal models is the gold standard tool for prion strain typing. Upon experimental transmission, strains show distinctive incubation periods and biochemical and neuropathological features maintained after sub-passage in the same species [4]. Although several studies indicate that the origin of strains is related to a specific conformation of PrP^Sc^, the molecular pathways underlying the pathogenesis and the phenotypic heterogeneity among strains are not fully understood [5,6,7].

Astrogliosis is one of the main pathological hallmarks of prion diseases. In fact, astrocyte reactivity, characterized by morphological changes and upregulation of glial fibrillary acidic protein (GFAP), a marker of astrocyte activation, has been widely observed in both natural hosts and experimentally infected animals [8,9,10]. Furthermore, several studies indicate that astrocytes are actively involved in prion pathogenesis [11,12,13], and their ability to accumulate and replicate PrP^Sc^ suggests an active role in prion propagation [14,15,16,17,18,19,20]. Nonetheless, the precise role of these glial cells and the factors triggering their reactivity is still debated [21,22].

Long regarded as a homogenous cellular population, astrocytes are highly heterogeneous. They differ in morphology and functions across distinct brain regions and sub-regions in rodents and humans [23,24,25,26]. Remarkably, independent studies demonstrated that different initiating injuries can elicit distinct astrocyte phenotypes, extending the concept of heterogeneity to reactive astrocytes in pathological conditions [27,28]. Several recent studies investigated the multifaceted involvement of astrocytes in prion diseases. For instance, recent evidence pointed out the influence of distinct factors, such as the brain region involved [29,30], the genotype backgrounds (*PRNP*) of the host [31], and the prion strain [19,31,32] on the astrocyte phenotype.

Elucidating the influence of prion strains on the reactive astrocyte phenotype may provide insights into the mechanisms triggering their reactivity and the consequences of this cellular response. The definition of these mechanisms is crucial for the development of new therapeutic strategies for these incurable conditions. Interestingly, other neurodegenerative diseases, like prion diseases, show clinicopathological variability, which has been explained as the existence of different strains. Thus, investigating the relationship between distinct strains/conformers and astrocyte phenotypes in prion diseases, and providing details on the pathogenesis of prion diseases, may be relevant for unraveling the involvement of astrocytes in other neurodegenerative diseases.

Until now, few studies have addressed the differential response of astrocytes to different prion strains, obtaining controversial results. In particular, some authors reported strain-specific expression levels of reactive astrocyte markers in sCJD-affected patients and mice experimentally infected with mouse-adapted strains [31,32]. Furthermore, an astrocyte cell line that differentially propagates distinct murine prions has been isolated [19]. Conversely, two independent studies demonstrated a common neuroinflammatory response in different scrapie strains, indicating a universal astrocyte phenotype regardless of the strain [9,33]. Noteworthy, most of the comparative studies on prion strains compared a few strains or panels of strains with similar phenotypes.

In the present work, we performed a comparative study on the involvement of astrocytes in rodent-adapted prion strains characterized by different origins, disease kinetics, and neuropathological phenotypes. In particular, we performed an in-depth study of strain-related morphology of astrocytes in different prion strains using the bank vole (*Myodes glareolus*), a wild rodent for which we demonstrated high susceptibility to several human and animal prion sources in the last two decades [34,35,36,37,38,39,40,41,42,43]. Of note, a recent in vitro study has presented an astrocyte-based primary glia cell assay from bank vole, which is infectible with distinct scrapie strains [20]. Here, by immunohistochemistry, we achieved a morphometric and morphological comparative study of reactive astrocytes of six vole-adapted strains isolated from human and animal prion isolates. In particular, we selected the mediodorsal thalamic nucleus (MDTN) as a commonly affected region in all strains. Interestingly, we found that astrocytes in MDTN reacted to different prion strains by acquiring variable and strain-specific morphologies, possibly depending on their relationship with PrP^Sc^ deposition.

## 2. Materials and Methods

### 2.1. Experimental Design

In order to compare reactive astrocytes in prion strains having diverse features and to avoid any host-dependent effect, it is necessary to propagate prion strains in the same animal model. Bank voles represent an ideal model, as they are susceptible to a wide variety of prion strains from different species and of different etiology. Indeed, we have shown that voles are susceptible to prion strains of infectious or idiopathic origin from both humans [34,35,40,42,44,45] and animals [36,38,39,43,46,47], as well as to human prion strains caused by PrP mutations [34,41,44,48], hence of spontaneous etiology. We have been working with this animal model for several years. We have selected two genetic lines of voles, either coding for methionine (BvM) or isoleucine (BvI) at PrP codon 109 [49]. For the present work, we selected BvI as they allow for widening the spectrum of strains, being susceptible and faithfully propagating some atypical forms that transmit less efficiently in BvM, such as atypical scrapie [43], VPSPr [35], and GSS [41,48,50,51]. Based on our previous and ongoing projects aiming at characterizing prion strains by transmission in voles, we thus selected six BvI-adapted prions strains derived from humans or different animal species, representing well-known idiopathic (sporadic CJD subtypes), infectious (classical scrapie and North American CWD), or genetic (GSS) prion diseases.

### 2.2. Prion Strains

As human-derived prion strains, we included three sCJD subtypes (MV1, MM2T, and VV2) and GSS-A117V, selected because they show distinct neuropathological phenotypes and incubation times after two passages in BvI. Sporadic CJD cases have been originally transmitted in BvM [34] and later in BvI (Rossi et al., 2017 and unpublished results). BvI-adapted (two passages) sCJD-MV1 [44] and sCJD-MM2T (unpublished results) derive from this first set of experiments. BvI-adapted sCJD-VV2 (two passages) derives from an ongoing and larger project involving more sCJD subtypes (unpublished results), while BvI-adapted GSS-A117V (two passages) derives from recently published experiments [41].

As animal-derived strains, we included classical scrapie (CS) and North American CWD (NA-CWD), known to have distinct neuropathological features and disease kinetics in BvI, with BvI-adapted NA-CWD being a remarkably fast replicating strain [39]. BvM efficiently propagates several different CS isolates [34,38], being particularly susceptible to a CS strain prevalent in Italian small ruminants [40,47]. We have previously shown that Italian scrapie isolates transmit very efficiently also in BvI [39], which prompted a larger strain typing study of CS involving BvM and BvI. BvI-adapted CS used in this study is still unpublished and derives from this project. Finally, we have previously shown that North American CWD isolates from different cervid species easily transmit in BvI and converge into a single BvI-adapted NA-CWD strain [36,39]. The BvI-adapted NA-CWD strain used in the present study derives from the third passage in BvI of a Canadian moose isolate [36].

Full experimental details and results of the transmission studies from which we obtained BvI-adapted sCJD-MM2T, sCJD-VV2, and CS will be reported elsewhere. Experiments involving animals adhered to the guidelines contained in the Italian Legislative Decree 26/2014, which transposed the European Directive 2010/63/UE on Laboratory Animal Protection. The experimental protocols were approved and supervised by the Service for Biotechnology and Animal Welfare of the ISS and were authorized by the Italian Ministry of Health (decree number 1119/2015-PR).

### 2.3. Samples

For this study, we selected paraffin-embedded brain tissues from terminally ill BvI from the six transmission experiments detailed above. In particular, we used brain tissues derived from second or third vole passages of sCJD-MV1 (*n* = 10), sCJD-MM2T (*n* = 6), sCJD-VV2 (*n* = 11), GSS-A117V (*n* = 12), CS (*n* = 8), and CWD (*n* = 12). Six mock-inoculated voles of ~500 days were similarly sampled as negative controls.

Brain sampling procedure has been previously reported for sCJD-MV1, GSS-A117V, and CWD [36,41,44]. The same sampling protocol was used for both previous works and the present paper. Briefly, voles were inoculated intracerebrally and culled with carbon dioxide at the terminal stage of the disease. The brain from each vole was removed and cut sagittally into two parts: one stored at −80 °C and one fixed in formol-saline.

Survival time was calculated as the interval between inoculation and culling or death.

### 2.4. Neuropathology and Lesion Profile

For neuropathological analysis and evaluation of spongiosis by lesion profiles, brain sections were cut at 6 μm and stained with hematoxylin and eosin.

For comparative purposes, in the present paper, we reported the lesion profiles of sCJD-MV1 and CWD, which have been previously published [36,44], while we determined the lesion profiles of the other strains using the same method [34,52].

The lesion profile was determined by evaluating the spongiform change and assigning a score from 0 to 5, depending on the severity of vacuolation, in nine grey-matter areas of the brain. Vacuolation scores were derived from at least six individual voles for strain and were reported as means ± standard error of the mean. All neuropathological assessments were performed blindly by two independent neuropathologists using Zeiss Axio Imager.M2 microscope.

### 2.5. Paraffin-Embedded Tissue Blot (PET-Blot)

Pet-blot was used for the evaluation of PrP^Sc^ distribution in the brains. We have previously shown the PET-blot images of three of six strains, namely sCJD-MV1, GSS-A117V, and CWD [36,41,44]. In this study, in order to compare the PrP^Sc^ distribution among all strains, we performed the PET-blot of previously published strains and those included in this work. For each strain, three vole brains were analyzed.

Brain sections of 6 μm were collected on 0.45-μm-pore nitrocellulose membranes (Schleicher & Schuell, Dassel, Germany) and dried at 37 °C for two days. After deparaffination and hydration, sections were treated with proteinase K (50 μg/mL) overnight at 55 °C. Next, proteins on the membranes were denatured using guanidine isothiocyanate 4 M for 15 min. After incubation with blocking solution for 60 min, sections were incubated with mouse antibody SAF84 (Alfatech; Auckland, New Zealand, 1:200) for 90 min. Following washes in TBS with 0.1% Tween-20, a rabbit anti-mouse alkaline phosphatase-conjugated antibody was applied for 90 min. After washing in TBST, membranes were adjusted to alkaline pH by incubating two times in NTM (Tris-HCl 100 mmol/L, NaCl 100 mmol/L; MgCl_2_ 50 mmol/L). The antibody reaction was visualized by formazan reaction using NBT/BCIP.

### 2.6. Immunohistochemistry

Brain sections of 4 μm were collected on Superfrost slides for GFAP staining and dried at 37 °C for 1 day. After deparaffination and hydration, sections were autoclaved for 30 min in 0.2% of citrate buffer solution at pH 6.2 at 121 °C. Next, endogenous peroxidases were blocked with a 3% hydrogen peroxide solution for 20 min, while non-specific antibody binding sites were blocked using 6% normal goat serum for 1 h. Immunohistochemical detection of GFAP was performed by incubating sections overnight at 4 °C with an anti-GFAP antibody (Thermo-Scientific, Waltham, MA, USA, 1:200). After washing in PBS with 0.1% Tween-20 and incubating with the appropriate biotinylated secondary antibody (goat anti-rabbit Vector Labs, Burlingame, CA 1:200), sections were treated with ABC Complex (Vector) for 1 h. Lastly, sections were stained with diaminobenzidine (Dako-Cytomation, Glostrup, Denmark) and counterstained with Mayer’s hematoxylin. Gfap-labeled sections designated for morphometric analysis were not counterstained. Each IHC run included positive and negative control sections.

### 2.7. Immunofluorescence

Sections of 4 μm were cut and collected on Superfrost slides to detect PrP^Sc^ in combination with GFAP. Briefly, sections were deparaffinated, rehydrated, and autoclaved in 0.2% of citrate buffer solution at pH 6.2 at 121 °C for 30 min. After immersion in a 3% hydrogen peroxide solution for 20 min, sections were incubated in 6% normal goat serum for 20 min. Anti-PrP^Sc^ antibody, SAF84 (Alfatech, Auckland, New Zealand, 1:200), 12B2 (Central Veterinary Institute, Lelystad, The Netherlands; 1:500), and anti-GFAP antibody (Thermo-Scientific, Waltham, MA, USA, 1:200) were used in combination and incubated overnight at 27 °C. Next, sections were washed with PBST and incubated with the appropriate fluorochrome–conjugated secondary antibodies for 1 h at 27 °C (Alexa Fluor 488 goat anti-mouse IgG2b Thermo-Scientific, Waltham, MA, USA, 1:300; Alexa Fluor 488 goat anti-mouse IgG Thermo-Scientific, Waltham, MA, USA, 1:250; Rhodamine (TRITC) Goat Anti-Rabbit IgG (H + L), Jackson Immunoresearch, Baltimore Pike, PA 1:150). Finally, to minimize the auto-fluorescence level in tissue sections, sections were treated with Vector^®^ TrueVIEW™ Autofluorescence Quenching Kit with DAPI. As suggested by suppliers, slides were evaluated within 48 h of mounting. Immunofluorescence was performed on three BvI brains for strain. For each brain, we analyzed three sections and for each section, two fields were evaluated. Brains were imaged using an Axio Imager.M2 microscope (Zeiss), operated by Zen software (Zeiss).

### 2.8. Cellular Area Measurement with Fiji Software

For cellular area measurement, GFAP-stained brain sections without hematoxylin counterstain were processed systematically, applying the newly developed protocol with Fiji software v2.0.0-rc-69/1.52p (https://fiji.sc, accessed on 11 November 2019). Astrocytes were sampled in a previously well-defined area of MDTN underneath the habenula. Astrocytes with an almost complete cellular shape, corresponding to a well-recognizable cellular body and at least three well-defined processes, were included in the current analysis. Astrocytes surrounding blood vessels, amyloid plaques, or vacuoles were excluded. Every single astrocyte selected was processed, converting the original DAB-stained image into an 8-bit grayscale image and then to a binary format with Bernsen’s thresholding method. Finally, the cellular area was quantified as the total number of pixels present in the outline cell shape of astrocytes, later transformed to squared micrometers (Figure S1). Some images were manually edited to close the shape and to reproduce the original cellular portrait. Where necessary, some pixels were cleared to separate ramifications pertaining to neighboring cells, while some pixels were added to join processes belonging to the selected cell. These steps were carefully done with the highest magnification of original and/or processed images. For each strain, we analyzed at least three vole brains and two serial sections for each brain. Only vole brains displaying a minimum of three astrocytes fulfilling the selection criteria were included in the analysis. Mean values of the cellular area of each single vole brain were reported as means ± standard deviation.

### 2.9. Statistical Analysis

We assessed the between-subject variability within each strain by one-way parametric analysis of variance (ANOVA) with the subject as a grouping factor (Table S1). In two strains, the variability between subjects was higher than the variability between astrocytes within subjects; thus, we performed the subsequent parametric analyses using subjects as statistical units.

In order to evaluate differences among strains, we performed one-way parametric ANOVA with strain as a grouping factor at six levels. Multiple comparisons between all pairs of strains were performed using the Tukey test to identify subgroups of strains characterized by significantly different cellular areas.

## 3. Results

### 3.1. Characterization of Vole-Adapted Strains

Human-derived strains showed survival times of 190 ± 8 days post-inoculation (d.p.i.) (*n* = 10) for sCJD-MV1, 246 ± 16 d.p.i. (*n* = 6) for sCJD-MM2T, 482 ± 55 d.p.i. (*n* = 11) for sCJD-VV2, and 107 ± 6 d.p.i. (*n* = 12) for GSS-A117V. In animal-derived strains, we observed survival times of 63 ± 8 d.p.i. (*n* = 8) in CS and 32 ± 3 d.p.i. (*n* = 11) in CWD.

**Figure 1:**
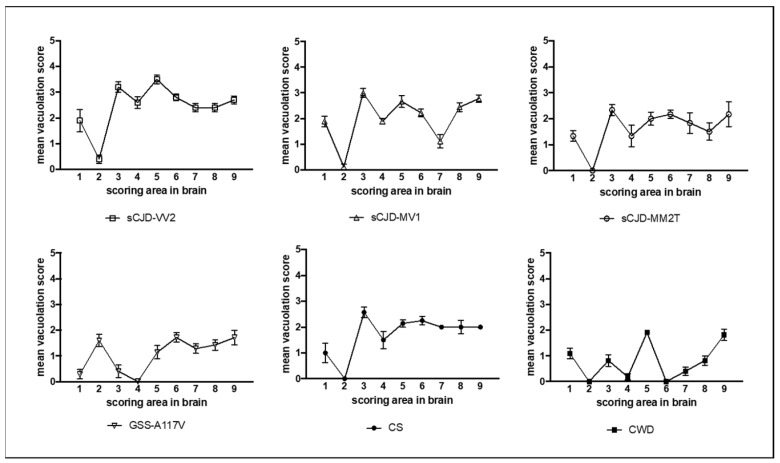
Lesion profiles of selected vole-adapted prion strains. Data points represent the mean (±SEM) of at least six voles per group. Brain-scoring positions are medulla (1), cerebellum (2), superior colliculus (3), hypothalamus (4), thalamus (5), hippocampus (6), septum (7), retrosplenial and adjacent motor cortex (8), and cingulate and adjacent motor cortex (9).

Spongiform degeneration was assessed using the lesion profile method. Lesion profiles showed that the severity and regional distribution of spongiform change varied depending on the strains (Figure 1). For example, sCJD-VV2 had the highest overall scores, particularly in the colliculus and thalamus, while CWD showed low-to-moderate degeneration limited to specific brain areas. The cerebellum was affected in GSS-A117V but mostly spared in all other strains. The hypothalamus was involved in all strains except for CWD and GSS-A117V. The hippocampus was free of spongiform degeneration only in CWD.

Besides allowing the quantitative evaluation of spongiform degeneration, the histopathological analysis also revealed some distinctive pathological characteristics of strains, such as thalamic atrophy in most voles affected with sCJD-MM2T (Figure S2a,b), small vacuoles scattering in the corpus callosum of GSS-A117V (Figure S2c) and widespread deposition of PrP^Sc^ amyloid plaques in several brain regions of sCJD-VV2 (Figure S2d).

**Figure 2:**
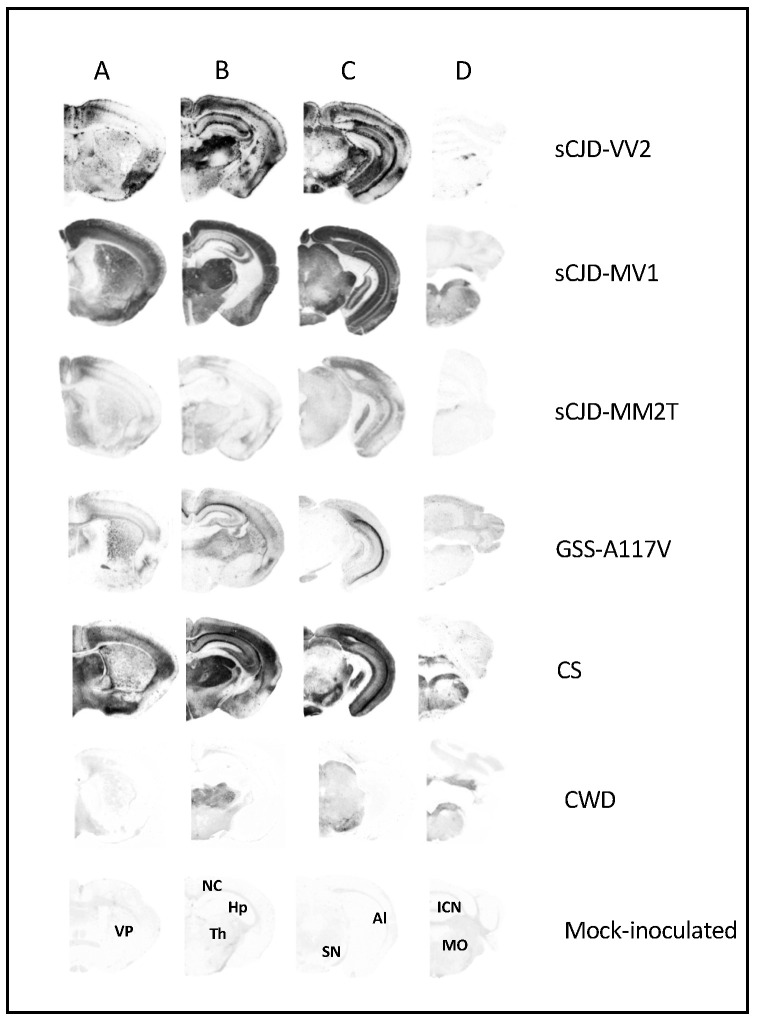
Regional distribution, by PET-blot, of PrP^Sc^ in selected vole-adapted prion strains. Coronal sections represent telencephalon (**A**), diencephalon (**B**), midbrain (**C**), and hindbrain (**D**). In the lower part of the figure, the labeled coronal sections of a negative control brain of 500 days are shown: VP, ventral pallidum; NC, neocortex; Hp, hippocampus; Th, thalamus; SN, substantia nigra; Al, alveus; ICN, interposed cerebellar nucleus; MO, medulla oblongata.

PET-blot analysis showed significant strain-dependent variations in both the intensity and neuroanatomical distribution of PrP^Sc^ deposition (Figure 2), which generally agreed with the distribution of spongiform degeneration, as shown by lesion profiles. A remarkable exception was the low level of PrP^Sc^ deposition detected in sCJD-MM2T compared to the relatively high degree of spongiform change in the same brain areas. Extensive and widespread plaques in the neuropil and large deposits along the alveus were observed in sCJD-VV2, while GSS-A117V was characterized by discrete immunolabelling of the alveus, as previously reported [41]. In sCJD-MV1, PrP^Sc^ predominated in cortices, thalamic nuclei, superior colliculus, lateral geniculate nucleus, and substantia nigra, in line with the distribution shown in Rossi et al., 2017 [44]. Interestingly, CS displayed intense immunolabelling of cortical and subcortical areas, with moderate labeling of caudate-putamen and grey matter of the midbrain, including periaqueductal grey matter. In contrast, CWD displayed intense labeling preferentially in subcortical areas and in the medulla.

Overall, these data showed that the selected strains were characterized by different neuropathological features and kinetics of replication. The phenotypical diversity observed among strains represented the effect of different pathogenetic processes, indicating that the selected strains represented an ideal panel to investigate the strain-specific response of astrocytes.

### 3.2. Comparison of Astrocyte Response in Vole-Adapted Strains

Strain-dependent variability of reactive astrocytes was investigated by morphometric and morphological approaches in all vole-adapted strains described above. By immunohistochemical detection of GFAP, we found prominent astrogliosis overlapping with both spongiform change and PrP^Sc^ deposition in all prion-affected brains. In contrast, we observed that immunolabelling in mock-inoculated vole brains, used as a negative control, was mainly located along the white matter tracts and around the blood vessels forming the blood-brain barrier.

Due to the morphological heterogeneity of astrocytes across different regions and sub-regions, we performed this comparative study within a selected brain area affected by all strains and identified during the neuropathological characterization. We thus chose the MDTN, as it showed a well-delimited structure and severe astrogliosis with the whole set of strains analyzed. In MDTN, we observed a variable response of reactive astrocytes that were evenly scattered in this nucleus (Figure S3). In particular, astrocytes showed significant differences in cellular size among strains.

**Figure 3:**
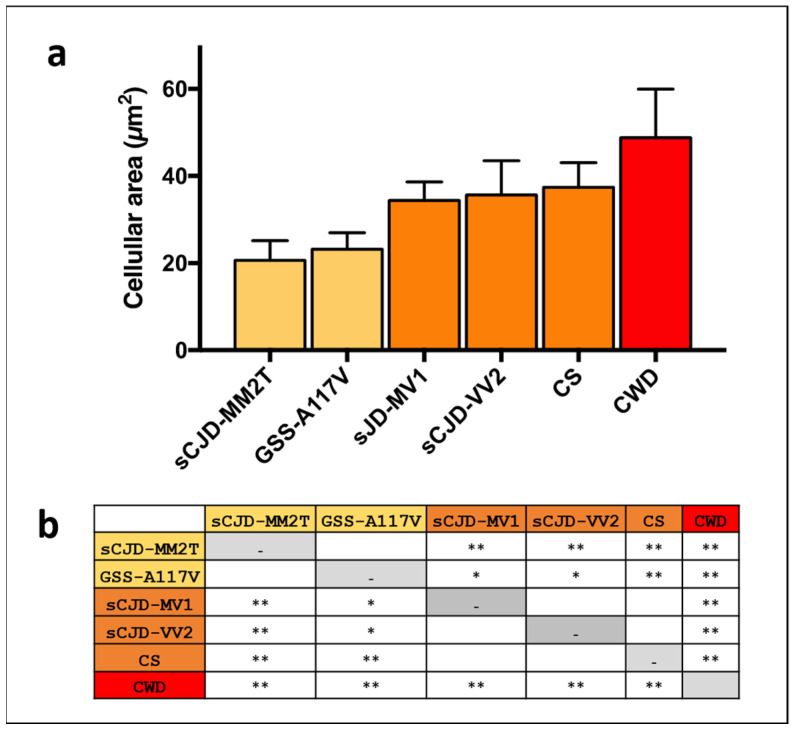
Cellular area of reactive astrocytes in prion strains investigated. In (**a**), measurements of the cellular area of reactive astrocytes are reported as mean value ± standard deviation. In (**b**), *p*-values obtained by Tukey test for multiple comparisons are reported (* *p*-value < 0.05; ** *p*-value < 0.01).

Thus, to investigate the variability of astrocytic size among strains, we measured the cellular area with Fiji software v2.0.0-rc-69/1.52p (https://fiji.sc, accessed on 11 November 2019) on the GFAP-stained astrocytes as described in M&M (Section 2.8). Interestingly, parametric ANOVA highlighted significant variability among strains (F(5.17) = 26.66, *p* < 0.0001). The Tukey test for multiple comparisons identified three subgroups, with strains not statistically different within each subgroup and significantly different from strains belonging to the other subgroups (Figure 3): subgroup A, including sCJD-MM2T and GSS-A117V, was characterized by the smallest astrocytes; subgroup B, including sCJD-MV1, sCJD-VV2, and CS, showed an intermediate cellular area; and subgroup C, consisting of CWD, showed the most prominent astrocytes (Figure 3).

**Figure 4:**
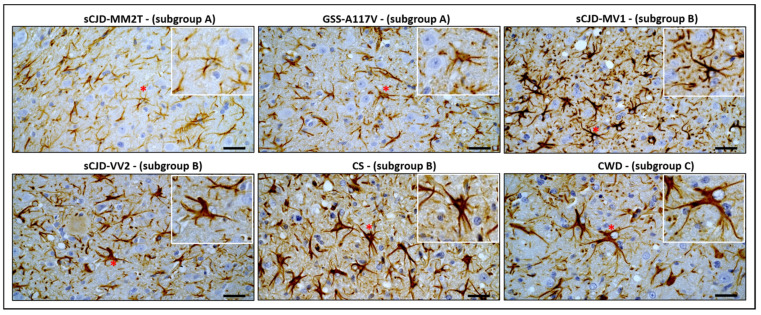
Reactive astrocytes in MDTN in selected vole-adapted strains. Comparison of morphological phenotypes of astrocytes observed in all strains analyzed. Magnifications of representative astrocytes extracted from the original image (red asterisks) are shown in inserts to highlight the morphological differences described in the text. For each strain, we analyzed four brains, and three sections were evaluated for each brain. Scale bars, 20 μm.

To investigate the morphological phenotype of reactive astrocytes in strains within the same subgroup, we performed a morphological evaluation of astrocytes considering the features of both cell bodies and processes (Figure 4). In subgroup A, astrocytes in sCJD-MM2T showed small cellular bodies and very thin processes, while those in GSS-A117V displayed short but thick processes (Figure 4). Slight morphological differences were also observed among astrocytes in subgroup B. Indeed, astrocytes showed long and thick processes in sCJD-MV1, and short and very thick processes in sCJD-VV2, while processes had variable thickness in CS (Figure 4). Finally, astrocytes in CWD were characterized by enlarged cellular bodies and very long and thick processes (Figure 4).

**Figure 5:**
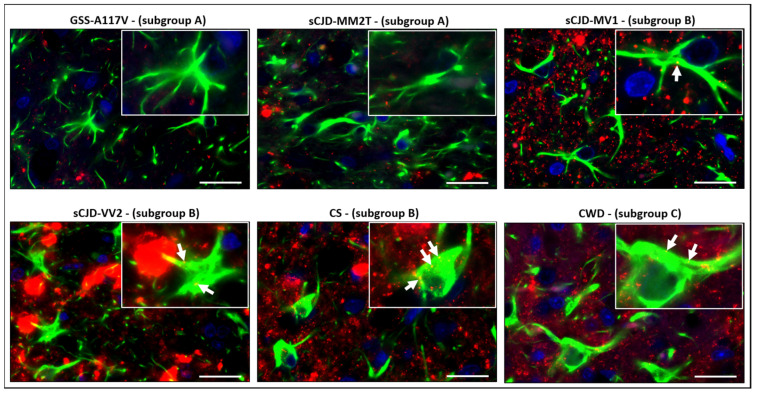
Double immunofluorescence staining of PrP^Sc^ and GFAP in MDTN of all selected strains. Immunofluorescence was performed on the brains of all strains using antibodies directed against PrP^Sc^ (red fluorescence) and GFAP (green fluorescence). GSS-A117V and sCJD-MM2T did not display astrocyte-associated PrP^Sc^ deposition. In contrast, astrocyte-associated PrP^Sc^ deposition was observed in sCJD-MV1, sCJD-VV2, CS, and CWD (yellow spots indicated by white arrows). Nuclei were counterstained with DAPI (blue). Scale bars, 20 μm.

To assess the relationship between astrocytes and PrP^Sc^ deposition, we performed a double immunofluorescence staining for both PrP^Sc^ and GFAP. By this technique, we observed astrocyte-associated PrP^Sc^ deposition in sCJD-MV1, sCJD-VV2, CS, and CWD but not in GSS-A117V and sCJD-MM2T (Figure 5). Thus, astrocyte-associated PrP^Sc^ deposition seemed to correlate with their cellular area, as the two strains which did not display astrocyte-associated PrP^Sc^ deposition were also those belonging to subgroup A and having the smallest reactive astrocytes among all strains.

## 4. Discussion

In order to investigate the influence of prion strains on the astrocyte phenotype, we compared the morphological response of astrocytes to prion infection in six vole-adapted strains with distinct origins and pathological features. Our results suggest that prion strains influence astrocyte phenotype in terms of cellular area, cellular shape, and their relationship with PrP^Sc^ deposits.

Morphometric and morphological approaches have been widely used to investigate the involvement of astrocytes in prion diseases [8,29,53,54,55,56,57,58]. Most of the published protocols for morphometric analysis include three-dimensional acquisitions or cellular reconstruction [57,58]. Here, we applied a rapid morphometric method consisting of a few simple steps to measure cellular area without three-dimensional reconstruction of astrocytes. In this context, morphometric data were used to assess the variability of astrocytes rather than to describe their morphological features finely. Indeed, qualitative evaluation of the morphology of reactive astrocytes highlighted other differences not detected by the quantitative approach. Thus, the simple morphometric approach used here must be combined with the morphological assessment of astrocytes to reveal fine differences among strains.

We speculated that the morphological variability of astrocytes observed in vole-adapted strains could be interpreted as distinct cellular subtypes; hence, each strain could be characterized by a distinctive and specific subtype of reactive astrocytes; or, alternatively, as different reactivity states, therefore, the morphological differences could represent different stages in a linear neuroinflammation process. Until now, only the latter hypothesis has been supported by studies that observed the asynchronous progression of the disease in different strains [9,33].

PrP^Sc^ accumulation appeared to trigger astrocyte reactivity; however, it remains to establish whether astrocyte reactivity is triggered by PrP^Sc^ accumulation within astrocytes or in the brain parenchyma. In this study, we found astrogliosis in all strains, despite astrocyte-associated PrP^Sc^ deposition was observed in four out of six strains. This evidence suggests that both PrP^Sc^ deposition in the brain-parenchyma- and astrocyte-associated PrP^Sc^ depostion could trigger astrocyte reactivity. Compared to neurons or microglial cells, which replicate or engulf prions, respectively, astrocytes can both replicate prions [15,19,20,59] and contribute to PrP^Sc^ uptake from extracellular space [60]. Therefore, the astrocyte-associated PrP^Sc^ deposition could result from both replication and uptake of prions, suggestive of a different role of astrocytes in prion pathogenesis.

Of note, we found a correlation between the size of astrocytes and their relationship with PrP^Sc^ deposition. In particular, strains characterized by the smallest astrocytes did not display astrocyte-associated PrP^Sc^ deposition, while strains with larger astrocytes showed astrocyte-associated PrP^Sc^ deposition. Other authors addressing the astrocyte response in a transgenic AD model found that astrocytes’ hypertrophic or atrophic appearance depends on the distance from the amyloid deposits [61]. It remains to elucidate whether the large size of astrocytes in vole brains depends on their relationship with PrP^Sc^ deposition.

Interestingly, we did not observe a correlation between the degree of spongiform change and the size of astrocytes. In fact, two strains, such as sCJD-MM2T and CWD, which showed a similar score of spongiform change in MDTN, displayed astrocytes with different sizes. Similarly, the survival time appeared to not influence the size of astrocytes. Indeed, strains grouped together for the size of astrocytes were characterized by significantly different survival times. For instance, CS and sCJD-VV2 displayed astrocytes with similar size, even though they induced disease in voles at two and sixteen months, respectively.

In line with the evidence demonstrating that morphological rearrangement of astrocytes implies a change in cellular functions [58,61,62,63,64], the morphological variability along with the differential relationship with PrP^Sc^ deposition suggest that astrocytes in voles could exert distinct functions during the pathogenesis of different strains. To well characterize the reactive state of astrocytes, further analyses of specific markers are needed. In bank vole, the neuropathological investigations are limited by the lack of species-specific reagents, thus new efforts are required to evaluate the most adequate approach for detecting vole astrocytic markers. In particular, we will evaluate the homeostatic, neuroprotective, and neurotoxic markers [11,32,65,66,67] to determine the phenotype of reactive astrocytes.

The role of reactive astrocytes in the pathogenesis of prions has been only recently investigated [12,68,69] and both beneficial and detrimental functions have been reported. The controversial results reported so far make it difficult to outline the therapeutic strategies targeting these cells [11,12,13,22,60]. In this context, our results on the heterogeneous response of astrocytes in distinct strains add an element to consider in developing new pharmacological treatments. This suggestion could also be extended to other neurodegenerative diseases in which strain variability has been observed, such as Alzheimer’s disease and tauopathies [70,71,72].

In conclusion, in this study, we highlighted for the first time the different morphological responses of astrocytes to distinct prion strains. Future studies will be performed to investigate the mechanisms underlying the morphological variability of astrocytes and the pathological effects of the heterogeneity presented here.

## Data Availability

Data is contained within the article or supplementary material.

## Supplementary Materials

The following supporting information can be downloaded at: https://www.mdpi.com/article/10.3390/biom13050757/s1, Figure S1: Progressive representation of Fiji protocol developed to measure the cellular area starting from a DAB-stained image. Figure S2: Specific neuropathological features of sCJD-MM2T, GSS-A117V, and sCJD-VV2. Figure S3: Immunohistochemical detection of GFAP in MDTN of selected vole-adapted prion strains. Table S1: Dataset of cellular areas of astrocytes in prion strains investigated.
