# Supplementary material for: Strain-Dependent Morphology of Reactive Astrocytes in Human- and Animal-Vole-Adapted Prions
Source: Biomolecules. 2023 Apr 27;13(5):757. doi: 10.3390/biom13050757 (PMC10216604; doi:10.3390/biom13050757)
Supplement: Supplementary file 1 [file biomolecules-13-00757-s001.zip › biomolecules-2312653-Supplementary materials.pdf]

Supplementary materials of:

Strain-Dependent Morphology of Reactive Astrocytes in Human- and Animal-Vole-Adapted Prions

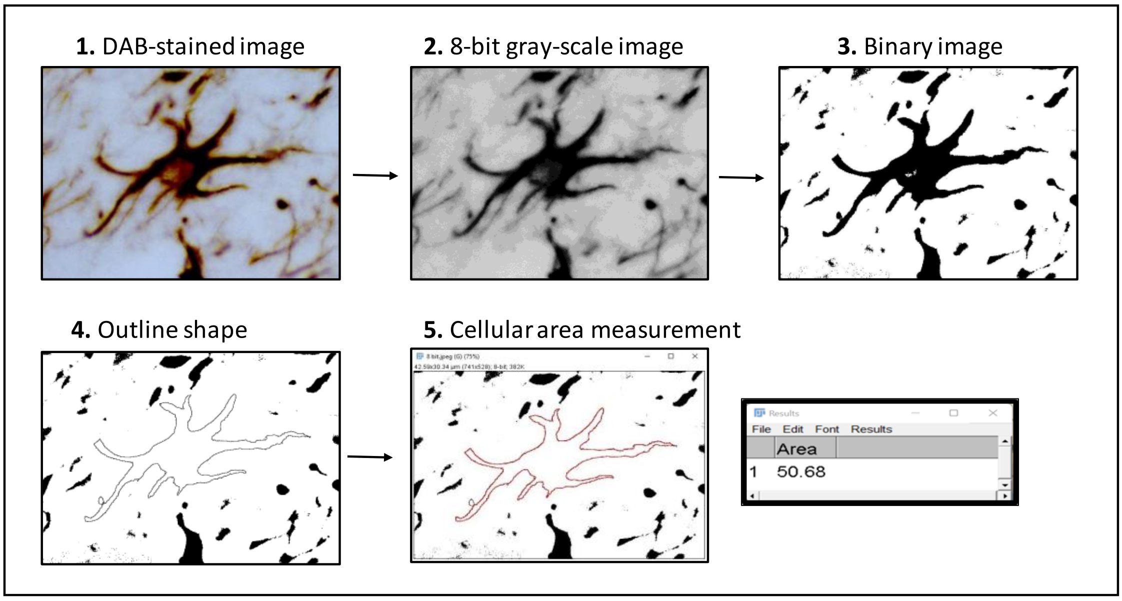

Figure S1: Progressive representation of Fiji protocol developed to measure the cellular area starting from a DAB-stained image.

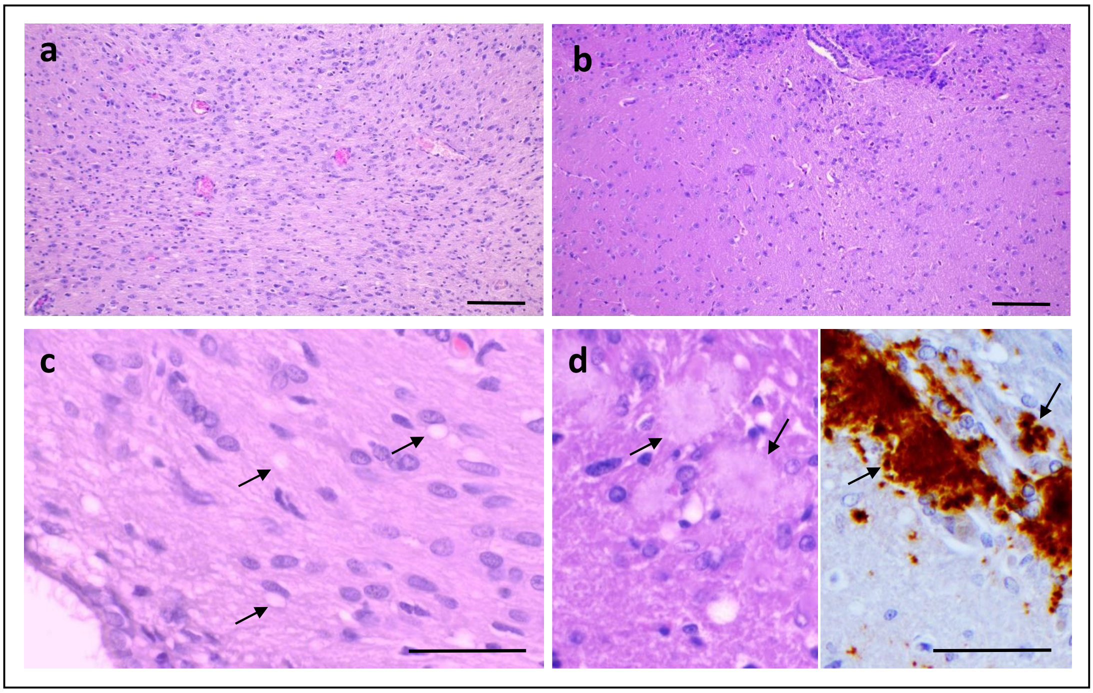

Figure S2 - Specific neuropathological features of sCJD-MM2T, GSS-A117V and sCJD-VV2. Haematoxylin and eosin staining highlighted thalamic atrophy in sCJD-MM2T (a) in comparison to the thalamus of mock-inoculated vole (b) (scale bars, 50 µm); (c) small vacuoles in corpus callosum of GSS-A117V (scale bar, 20 µm); (d) (d) panel with haematoxylin and eosin (left) and immunohistochemistry (right) of plaques accumulated in the alveus of vole infected with sCJD-VV2 (scale bar, 20 µm).

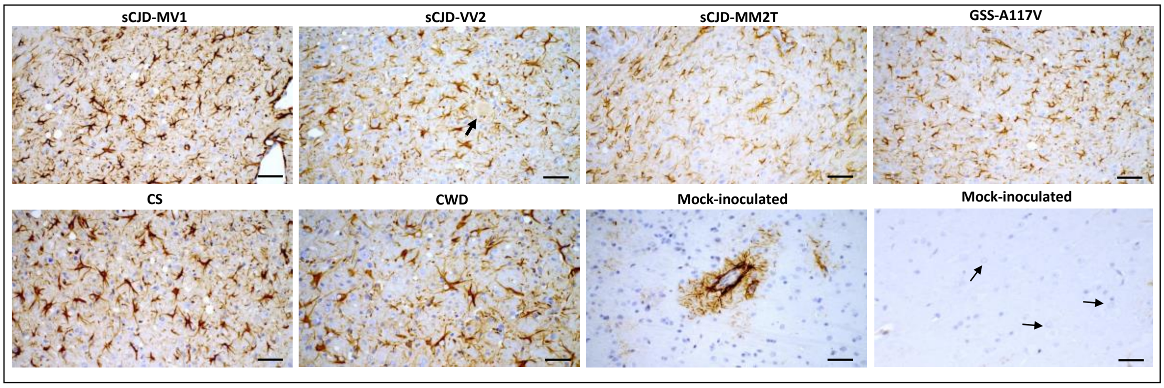

Figure S3 - Immunohistochemical detection of GFAP in MDTN of selected vole-adapted prion strains. Prominent astrogliosis affected all prion-infected vole brains. In sCJD-VV2, amyloid plaque encircled by few astrocytes is detected in the neuropil (arrow). In the thalamus of mock-inoculated voles, few astrocytic processes surrounding blood vessels are visible, while any GFAP immunolabelling is associated with astrocyte cellular bodies scattered within thalamic parenchyma. Scale bars, 20 µm.

Strain	Vole ID	Number of astrocytes	Mean of cellular area (µm^2)	Standard Deviation	Prob>f (Anova)
sCJD-MM2T	#1	12	23.85	3.89	0.0016
	#2	4	18.81	3.14	
	#3	9	17.46	3.33	
GSS-A117V	#1	15	23.55	4.20	0.3001
	#2	5	20.70	3.20	
	#3	6	23.67	3.44	
sCJD-MV1	#1	10	32.92	4.28	0.0529
	#2	11	34.62	3.79	
	#3	6	38.38	4.20	
	#4	8	32.95	3.53	
sCJD-VV2	#1	4	31.34	1.30	0.0124
	#2	5	39.33	8.36	
	#3	7	40.22	6.81	
	#4	4	27.43	3.33	
CS	#1	21	38.85	5.29	0.1889
	#2	5	35.09	5.78	
	#3	4	33.29	3.17	
	#4	12	35.98	6.06	
	#5	7	39.58	5.92	
CWD	#1	10	45.26	4.70	0.1183
	#2	6	49.54	8.89	
	#3	3	57.57	11.53	
	#4	3	50.31	7.75	

Table S1 – Dataset of cellular areas of astrocytes in prion strains investigated. Table includes: i) number of animals per strain; ii) astrocytes per vole analyzed for each strain; iii) the mean values ± standard deviation of the cellular area obtained from every single animal; iv) and results of one-way parametric ANOVA. Medium-high consistency was observed among voles inoculated with the same strain, except for sCJD-VV2 and sCJD-MM2T, for which between-subject variability was found.
